# Vitreous Hemorrhage in Posterior Uveal Melanocytoma: Two Case Reports

**DOI:** 10.1155/crop/5126550

**Published:** 2025-01-22

**Authors:** Lauren B. Yeager, Chloe Y. Li, Dmitry Bogolmony, Larisa Debelenko, Brian P. Marr

**Affiliations:** ^1^Department of Ophthalmology, New York-Presbyterian/Columbia University Irving Medical Center, New York, New York, USA; ^2^Department of Pathology, New York-Presbyterian/Columbia University Irving Medical Center, New York, New York, USA

**Keywords:** choroidal melanocytoma, ciliary body melanocytoma, malignant transformation, uveal melanocytoma, vitreous hemorrhage

## Abstract

**Purpose:** We describe the diagnostic challenge by melanocytomas originating from locations other than the optic nerve.

**Methods:** This is a retrospective case report of two patients who presented with vitreous hemorrhage with underlying melanocytomas.

**Results:** Both patients had hemorrhage obscuring indeterminant uveal masses; fine-needle aspiration biopsies confirmed melanocytoma with necrosis and atypia and, in one case, concern for malignant transformation.

**Conclusion:** Melanocytomas are rare, benign melanocytic lesions that resemble nevi. In contrast to optic nerve melanocytoma, those involving the choroid and ciliary body lack specific clinical characteristics. Vitreous hemorrhage is an underrecognized complication, and uveal melanocytoma must be included in the differential diagnosis of vitreous hemorrhage with associated ciliary body or choroidal mass. Biopsy is required for definitive diagnosis and to identify malignant transformation of the lesion, a rare but possible occurrence.

## 1. Introduction

Melanocytomas are dark brown to black pigmentary tumors related to benign nevi. They typically occur in the optic nerve, but uveal melanocytoma is well documented. Unlike optic nerve melanocytoma which has classic clinical findings, posterior uveal melanocytoma can appear indistinguishable from other melanocytic tumors, including nevi and melanoma. Diagnosis can be challenging and requires tissue confirmation in suspicious cases. Herein, we describe two cases of uveal masses with accompanying vitreous hemorrhage that were diagnosed with fine-needle aspiration biopsy as melanocytoma with atypia, necrosis, and, in one case, concern for malignant transformation. The pathophysiology of vitreous hemorrhage in cases of melanocytoma has not been well established in the literature.

## 2. Case Report

### 2.1. Case 1

**Figure 1:**
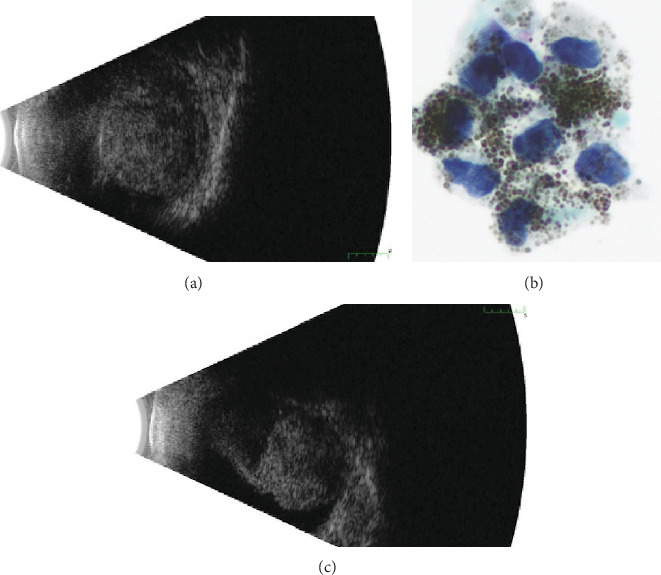
Ultrasonography and cytopathology in Case 1. (a) A 13.5 × 14.1 × 9‐mm ciliary body mass involving the choroid with vitreous hemorrhage on ultrasonography. Vascular pulsations were present. (b) Fine-needle aspirate of the right ciliary body mass showing a small cluster of polyhedral lesional cells with abundant cytoplasm filled with large spherical melanin granules obscuring the nuclei. The nuclei are somewhat enlarged but without prominent nucleoli. Cellular smear, original magnification ×600, Papanikolaou's stain. (c) At 1-year follow-up, there was a mass versus consolidated hemorrhage that significantly decreased in size from presentation.

An 80-year-old male was referred with progressive decreased vision over 9 months and a choroidal mass of the right eye. Past medical history was significant for cutaneous melanoma, and past ocular history included cataract extraction, age-related macular degeneration, and glaucoma. On examination, vision was hand motion in the right eye and 20/100 in the left eye. Intraocular pressure was 26 in the right eye and 12 in the left eye. In the right eye, there was diffuse hyphema and dense vitreous hemorrhage obscuring the view to the fundus. The left eye was normal. Ultrasonography on the right eye demonstrated a 13.5 × 14.1 x 9 mm ciliary body mass with heterogeneous echogenicity involving the choroid (Figure 1(a)). Intrinsic vascularization and subretinal fluid were present. There was no evidence of extraocular extension. Leading diagnoses included ciliary body melanoma, adenoma or adenocarcinoma, and peripheral exudative hemorrhagic chorioretinopathy. Fine-needle aspiration biopsy (FNAB) of the mass was performed and demonstrated small clusters of heavily pigmented polyhedral cells with large spherical melanocytic granules and relatively uniform bland nuclei (Figure 1(b)). Few nuclei displayed prominent nucleoli concerning for malignant transformation. The cells were positive for human melanoma black-45 (HMB-45), confirming the melanocytic differentiation of the lesion. Signs of necrosis were present. Based on these pathologic findings, the lesion was diagnosed as melanocytoma with focal atypia based on characteristic cytological features and rare cells with prominent nucleoli. Uveal melanoma, which was in differential diagnosis, usually contains finely dispersed melanin and shows prominent cellular atypia, mitoses, and extensive necrosis. Fortunately, none of these findings were present in this case, making uveal melanoma an unlikely diagnosis. The tumor decreased in thickness over the next year with observation (Figure 1(c)).

### 2.2. Case 2

**Figure 2:**
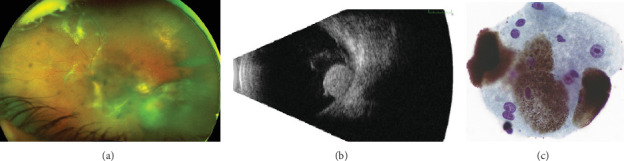
Multimodal imaging and cytopathology in Case 2. (a) Fundus photograph shows an amelanotic mass in the inferotemporal periphery with scattered retinal exudates, subretinal fluid, and overlying vitreous hemorrhage. (b) Ultrasonography of a 5.0 × 5.8‐mm choroidal mass at presentation. (c) Fine-needle aspirate of the left ciliary body mass (Case 2) showing a small cluster composed of differently pigmented lesional cells with low nucleo/cytoplasmic ratio. Large spherical melanin granules obscure the nuclei of some cells. In other less pigmented cells, the nuclei are enlarged and show prominent nucleoli (atypical features). Cellular smear, original magnification ×600, Papanikolaou's stain.

A 58-year-old female was referred to the ocular oncology service with vitreous hemorrhage and a choroidal mass of the left eye. Her medical history was significant for sickle cell trait, hypertension, and diabetes mellitus. On the exam, visual acuity was 20/30 in the right eye and count finger vision at 6 feet in the left eye. Intraocular pressure was normal. The right eye examination was within normal limits. The left eye had a vitreous hemorrhage and inferotemporal mass with accompanying hemorrhage and exudate (Figure 2(a)). Ultrasonography confirmed a 5.0 × 5.8 mm mass with high internal reflectivity and accompanying subretinal fluid (Figure 2(b)). Magnetic resonance imaging was deferred as it was of limited diagnostic value at that time. Fluorescein and indocyanine green angiography were also deferred in the setting of vitreous hemorrhage. There was no intrinsic vascularization or extraocular extension. The most likely diagnosis at the initial visit was vasoproliferative tumor, given its associated exudates, hemorrhage, lack of vascularity, and high internal reflectivity on the B-scan. Photodynamic therapy (PDT) was performed in the outpatient setting using an injection of 15 mg verteporfin and laser with a 7.8 mm spot size and 166 ms duration. Two months later, her vision improved to 20/400. The subretinal fluid and superior exudate surrounding the mass improved, but she had worsening vitreous hemorrhage. Repeat ultrasonography showed minimal enlargement of the mass. At this point, FNAB was performed for confirmatory diagnosis and showed a melanotic lesion composed, similarly to Case 1, of single and clustered heavily pigmented polyhedral cells with large spherical melanin granules frequently obscuring the nuclei (Figure 2(c)). The cells were characterized by a low nuclear-cytoplasmic ratio, consistent with a benign diagnosis of melanocytoma. However, cellular atypia and prominent nucleoli within the sample raised concern for eventual malignant transformation. Gene expression profiling using the DecisionDx-Melanoma panel (Castle Biosciences, Inc.), which tests for 31 genes that can stratify cutaneous melanoma patients' risk for metastasis, was performed prior to plaque placement. This patient had a Class 1A result, indicating the lowest risk for metastasis. She also had a negative PRAME (preferentially expressed in uveal melanoma) mutation result, further supporting a low risk for metastasis. After a discussion with the patient, the lesion was treated with I-125 plaque brachytherapy.

## 3. Discussion

Melanocytoma classically appears at the optic nerve head as dark brown to gray–black lesions at the peripapillary choroid or anterior optic cup. Yet, unlike optic nerve melanocytoma, choroidal and ciliary body melanocytoma do not possess highly specific features, and there are no reliable clinical diagnostic criteria [1]. Documented cases of enucleation of benign melanocytoma misdiagnosed clinically as melanoma exist.

The benign lesions are typically without complications. Melanocytoma can undergo acute ischemic necrosis, releasing pigment into the vitreous and anterior chamber which can lead to increased intraocular pressure, secondary glaucoma, and at times, profound vision loss. In rare instances, melanocytoma undergoes malignant transformation into melanoma. This occurs in 1-2% of optic nerve melanocytoma, but the incidence in uveal cases is unknown [2].

A PubMed search was performed to identify all documented cases of biopsy-proven, posterior uveal melanocytoma involving the choroid and ciliary body. The search terms included (melanocytoma) AND (uvea OR choroid OR ciliary body). Cases without histopathology were excluded, as were papers that could not be translated into English. In total, 80 cases were identified. Three cases of posterior uveal melanocytoma with associated vitreous hemorrhage were described [3–5].

Kurli et al. described a 37-year-old female with a vitreous hemorrhage and choroidal mass. FNAB revealed a melanocytoma. Despite the benign diagnosis, the patient had loss of vision, elevated intraocular pressure, and pain, and the eye was enucleated. Histology confirmed a necrotic melanocytoma with atypia and malignant transformation [3]. Sia et al. described a case of a 68-year-old female with blurred vision and a pigmented choroidal mass. FNAB was performed; the results were inconclusive. After a period of observation, growth, vitreous hemorrhage, and retinal detachment were noted. Repeat cytology revealed rare atypia. Due to concern for melanoma, plaque brachytherapy was performed followed by eventual enucleation in the setting of a blind, painful eye. Pathology confirmed a melanocytoma of the choroid and ciliary body with extrascleral extension and necrosis without malignant transformation [5]. Rubin described a case of a 49-year-old female followed for a pigmented choroidal lesion that developed associated subretinal neovascularization and vitreous hemorrhage. The eye was enucleated for concern of choroidal melanoma. Diagnosis proved a melanocytoma with a nasal break in Bruch's membrane [4].

The mechanism of vitreous hemorrhage in posterior melanocytoma is unknown. Abnormal vessel growth and vessel behavior are known characteristics of melanoma, and it is suggested that angiogenesis is an important part of early development in uveal melanomas. [6] Four of the five cases outlined had evidence of atypia. We speculate that the presence of atypia or malignant transformation may have introduced angiogenic factors and the development of early blood vessel formation, which may have predisposed the lesion to a vitreous hemorrhage. This suggests a possible association between vitreous hemorrhage and atypia; however, further study is needed.

In the case of Rubin, there are two potential explanations for the vitreous hemorrhage. First, there was evidence of choroidal neovascularization. While this is not typically described in melanocytoma, choroidal neovascularization has been reported in choroidal nevi and can lead to vitreous heme [7]. Secondly, there was evidence of a break in Bruch's membrane. Likely, the mechanism is the same as which has been hypothesized in choroidal melanoma: Tumor herniates through Bruch's membrane and is compressed. The focal tourniquet effect leads to diminished venous return and venous stagnation, vascular dilatation, and eventual vessel rupture [8].

## 4. Conclusions

These cases provide insight into the clinical picture of a rare and challenging clinical entity and introduce vitreous hemorrhage as a rare complication of posterior uveal melanocytoma occurring in 6% of documented cases. Melanocytoma should be included in the differential diagnosis of vitreous hemorrhage with associated posterior uveal mass. Research on melanocytoma is challenging given the need for pathologic tissue and that most lesions do not ultimately get biopsied. However, it is important to further our knowledge of these benign lesions to avoid misdiagnosis and unnecessary invasive treatments.

## Data Availability

All data relevant to this case are included in this publication.
